# Mechanism and functional role of the interaction between CP190 and the architectural protein Pita in *Drosophila melanogaster*

**DOI:** 10.1186/s13072-021-00391-x

**Published:** 2021-03-22

**Authors:** Marat Sabirov, Olga Kyrchanova, Galina V. Pokholkova, Artem Bonchuk, Natalia Klimenko, Elena Belova, Igor F. Zhimulev, Oksana Maksimenko, Pavel Georgiev

**Affiliations:** 1 Department of the Control of Genetic Processes, Institute of Gene Biology, Russian Academy of Sciences, 3 4/5 Vavilov St., Moscow, 119334 Russia; 2 Center for Precision Genome Editing and Genetic Technologies for Biomedicine, Institute of Gene Biology, Russian Academy of Sciences, 34/5 Vavilov St., Moscow, 119334 Russia; 3 Institute of Molecular and Cellular Biology of the Siberian Branch of the Russian Academy of Sciences (IMCB RAS), Novosibirsk, Russia

**Keywords:** Chromatin insulator, Bithorax, Abd-B, BTB domain, Domain boundary, Open chromatin

## Abstract

**Background:**

Pita is required for Drosophila development and binds specifically to a long motif in active promoters and insulators. Pita belongs to the Drosophila family of zinc-finger architectural proteins, which also includes Su(Hw) and the conserved among higher eukaryotes CTCF. The architectural proteins maintain the active state of regulatory elements and the long-distance interactions between them. In particular, Pita is involved in the formation of several boundaries between regulatory domains that controlled the expression of three *hox* genes in the Bithorax complex (BX-C). The CP190 protein is recruited to chromatin through interaction with the architectural proteins.

**Results:**

Using in vitro pull-down analysis, we precisely mapped two unstructured regions of Pita that interact with the BTB domain of CP190. Then we constructed transgenic lines expressing the Pita protein of the *wild-type* and mutant variants lacking CP190-interacting regions. We have demonstrated that CP190-interacting region of the Pita can maintain nucleosome-free open chromatin and is critical for Pita-mediated enhancer blocking activity in BX-C. At the same time, interaction with CP190 is not required for the in vivo function of the mutant Pita protein, which binds to the same regions of the genome as the wild-type protein. Unexpectedly, we found that CP190 was still associated with the most of genome regions bound by the mutant Pita protein, which suggested that other architectural proteins were continuing to recruit CP190 to these regions.

**Conclusions:**

The results directly demonstrate role of CP190 in insulation and support a model in which the regulatory elements are composed of combinations of binding sites that interact with several architectural proteins with similar functions.

**Supplementary Information:**

The online version contains supplementary material available at 10.1186/s13072-021-00391-x.

## Introduction

The development of modern approaches for the study of genome architecture, including chromosome conformation capture methods, coupled to high-throughput sequencing (Hi-C) and high-resolution microscopy techniques has revealed the hierarchical organization of genome [1, 2]. Chromosomes are composed of discrete sub-megabase domains, called topologically associated domains (TADs) [3–5]. In genomes, regulatory elements, including enhancers, promoters, insulators, and silencers, actively interact with each other, which determines the correct and stable level of gene expression [6, 7]. The boundaries between TADs delineate specific genomic regions, and more effective interactions between regulatory elements occur within these regions than between different regions [8]. According to the generally accepted model, the cohesin complex, which is retained at CTCF protein binding sites, plays a primary role in the formation of chromatin loops in mammals [9]. Auxiliary roles in the organization of specific interactions between enhancers and promoters have been assigned to the proteins LBD1, yin yang 1 (YY1), and ZF143 [10–13]. Because the LBD1 protein is the only one of these proteins to contain a well-described homodimerization domain [14], how specific interactions between enhancers and promoters occurs remains unclear.

In *Drosophila*, we suggested the existence of a large family of architectural proteins, which typically contain N-terminal homodimerization domains and arrays of the zinc-finger Cys2-His2 (C2H2) domains [15–22]. The specific interactions that occur between the N-terminal domains of architectural proteins can support selective distance interactions between regulatory elements. Pita belongs to a large family of architectural proteins that feature zinc finger-associated domains (ZADs) at the N-terminus [21, 23]. Investigations of three architectural proteins, Pita, Zw5, and ZIPIC, showed that the ZAD domains form only homodimers and support specific distance interactions between sites bound by the same architectural protein [17]. The 683 aa Pita protein contains an N-terminal ZAD domain (17–93 aa) and a central cluster, consisting of 10 C2H2 zinc-finger domains (286–562 aa) [24, 25]. Pita is an essential *Drosophila* protein, and the strong hypomorph *pita* mutants die during the larval stage [24, 26].

Pita binds to a large 15-bp consensus site that is frequently found in gene promoters and intergenic regulatory elements, including boundary/insulator elements in the Bithorax complex (Bx-C) [19, 25]. The Bithorax complex (BX-C) contains three homeotic genes, *Ultrabithorax* (*Ubx*), *abdominal-A* (*abd-A*), and *Abdominal-B* (*Abd-B*), which are responsible for specifying the parasegments (PS5 to PS13) that comprise the posterior two-thirds of the fly segments [27–29]. The expression of each homeotic gene in the appropriate parasegment-specific pattern is controlled by independent *cis*-regulatory domains that are separated by boundaries. For example, the regulatory domains *iab-5*, *iab-6*, and *iab-7*, determine the expression of *Abd-B* in the abdominal segments A5, A6, and A7, respectively. The *Mcp*, *Fab-6*, *Fab-7*, and *Fab-8* boundaries ensure the autonomous function of *iab* domains [30–37]. Pita binds to *Fab-7* and *Mcp* and is required for their boundary activities [19, 20, 38]. Five Pita binding sites can replace the *Fab-7* boundary in blocking the cross-talk of the *iab-6* and *iab-7* regulatory domains [19].

Previously, Pita was found to interact with CP190 [25], which is also known to bind several other C2H2 architectural proteins, including dCTCF and Suppressor of hairy wing [Su(Hw)] [18, 25, 39–42].

Here, we studied the interaction mechanisms between Pita and CP190. Two domains that interact with the BTB domain of CP190 were mapped in Pita. The recruitment of CP190 is required for the chromatin opening and insulator functions of Pita. However, mutant flies that express Pita lacking the CP190 interaction region display normal viability and *wild-type* (*wt*) phenotype, demonstrating that these activities are not essential for Pita functions in vivo.

## Results

### Mapping regions within the Pita protein that interact with the BTB domain of CP190

**Fig. 1.**
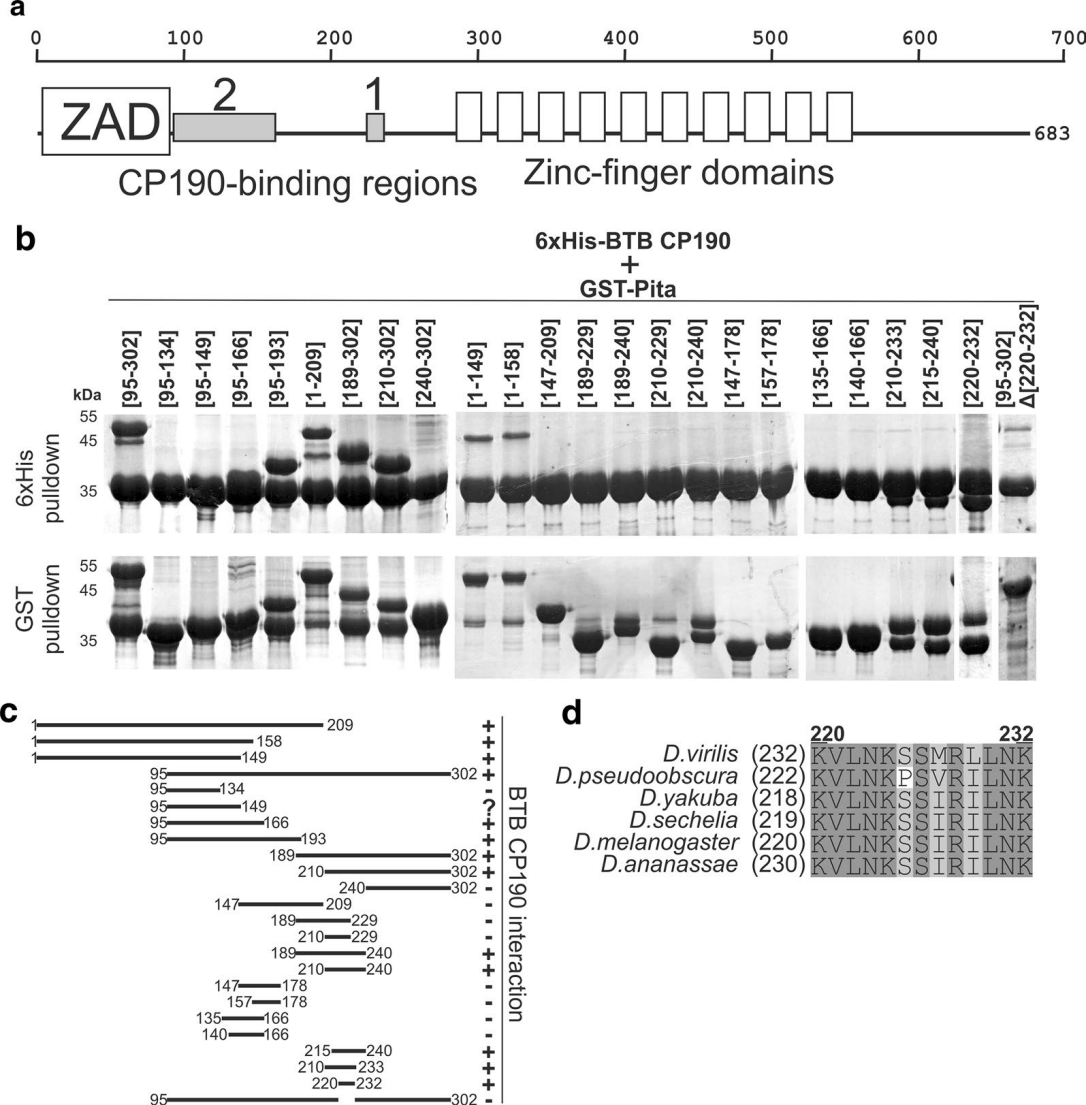
Mapping the CP190-interacting regions in the Pita protein. **a** Schematic representation of full-length Pita protein showing the CP190-binding regions (gray boxes). The positions of the amino acid residues are shown at the top of the panel. **b** GST- and 6×His-pull-down of GST-fused Pita protein fragments co-expressed with the thioredoxin-6×His-fused CP190 BTB domain. The positions of the amino acids are given in square brackets. **c** Schematic summary of the pull-down results. **d** Multiple sequence alignment of the CP190 BTB-domain-interacting peptide in Pita protein from various *Drosophila* species shows the high conservation of hydrophobic and positively charged residues. Residue numbers above the alignment are for *D. melanogaster* Pita protein.

To understand the interaction mechanism between the architectural protein Pita and the BTB domain of CP190, we attempted to precisely map the interaction regions in Pita. Previously, we found that the BTB domain of CP190 interacted with the 95–302 aa region of Pita, which was mapped between the ZAD and the C2H2 cluster [25]. We used bacteria to express overlapping glutathione *S*-transferase (GST)-fusion peptides that covered the 95–302 aa region of Pita. The borders of the deletion derivatives were set according to conserved blocks of amino acids in Pita protein from various Drosophila species. The obtained GST-peptides were tested for interactions with the CP190 BTB domain, fused with 6×His, in a pull-down assay (Fig. 1a). This process allowed us to map two binding regions between 95–165 aa and 220–232 aa (Fig. 1b, c). Interestingly, the deletion of 220–232 aa, which was defined as a 13 aa core, resulted in the complete loss of interaction between the 95–302 fragment and BTB in a pull-down assay, even though this protein fragment still contained the second binding region. The 13 aa core was predicted to be unstructured, but it contains several conserved hydrophobic residues (Fig. 1d).

Taken together, these results showed that the BTB domain interacts with the 95–165 aa region and the 13 aa core, whose sequences have no obvious homology. The 95–165 aa region appeared to stabilize the interaction between the BTB domain and the 13 aa core.

**Fig. 2:**
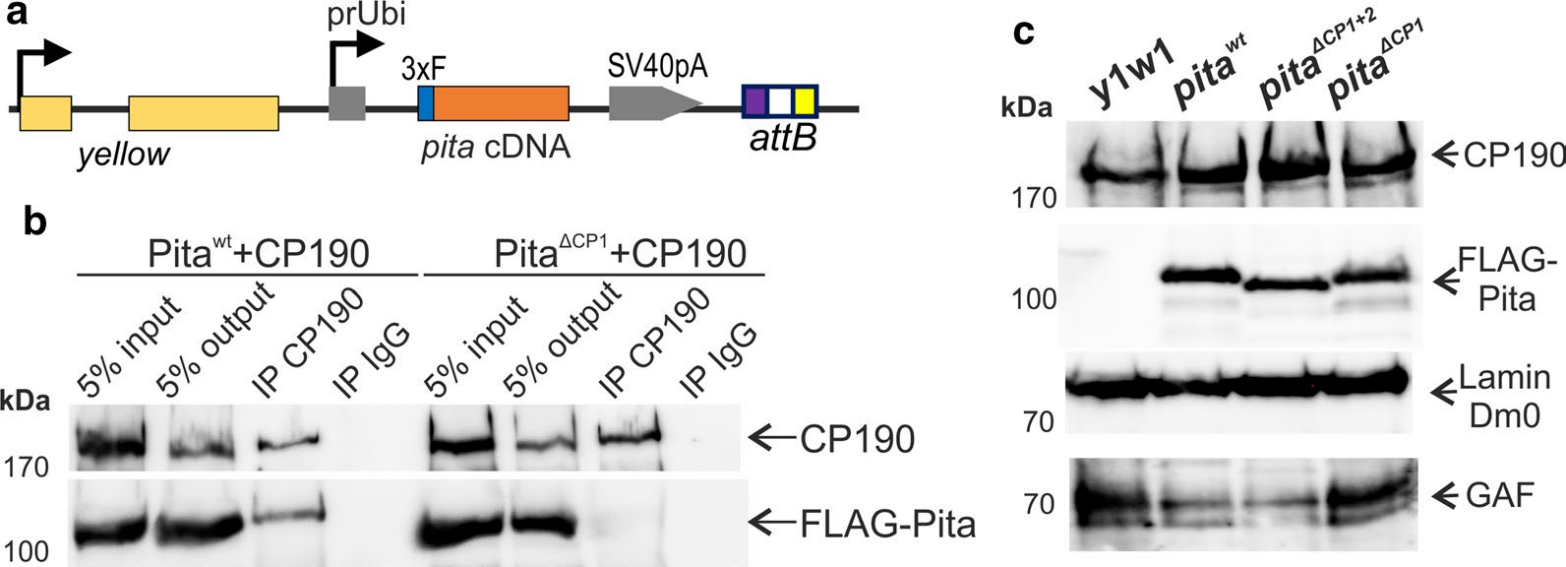
Mutations in the *pita* gene. **a** A schematic showing the constructs used to express wild-type and mutant variants of Pita in transgenic *Drosophila* lines. **b** Co-immunoprecipitation of CP190 with wild-type and CP190-interacting region-deleted Pita protein fused with 3×FLAG in S2 cells. Protein extracts from Drosophila S2 cells co-transfected with 3×FLAG-Pita and CP190 plasmids were immunoprecipitated with antibodies against CP190 (using nonspecific IgG as a negative control), and the immunoprecipitates (IP) were analyzed by western blotting for the presence of FLAG-tagged Pita proteins. The quality of immunoprecipitation was controlled by western blotting for the presence of CP190 protein. “Input” refers to samples of the initial protein extract; “output” refers to the supernatant after the removal of the immunoprecipitate (IP). **c** Western blot analysis (10% SDS-PAGE) of protein extracts from transgenic flies expressing wild-type and mutated variants of Pita

To better understand the functional significance of the interaction between Pita and CP190, we deleted the 13 aa core that is necessary for Pita to bind with CP190 in vivo (Pita^ΔCP1^). The Pita^*wt*^ and Pita^ΔCP1^ proteins were tagged with 3×FLAG (Fig. 2a) and co-expressed with CP190 in S2 cells (Fig. 2b). The mutant Pita^ΔCP1^ did not interact with CP190, in contrast with the Pita^*wt*^ protein. This result confirmed the critical role played by the 13 aa domain in the interaction between Pita and CP190 in vivo.

### The CP190-interacting domain in Pita is not essential for its role in *Drosophila* development

To understand the functional roles of the 13 aa core (CP1) and the 95–165 aa regions (CP2) in Pita, we used previously described strong hypomorph mutations in the *pita/spdk* gene: *pita*^*02132*^ and *pita*^*k05606*^ (Bloomington stock numbers 11179 and 10390, respectively). Pita protein is essential for early *Drosophila* development and mitoses, and homozygotes carrying the null mutation die at the larval stage [24, 26]. Transgenes expressing Pita^*wt*^-FLAG, Pita^ΔCP1^-FLAG, or Pita^ΔCP1+2^-FLAG under control of the Ubi-p63E promoter (*Ubi-Pita*^*wt*^*, Ubi-Pita*^ΔCP1^, and *Ubi-Pita*^ΔCP1+2^) (Fig. 2a) were inserted into the same 86Fb region on the third chromosome, using a φC31 integrase-based integration system [43]. Western blot analysis showed that Pita^*wt*^-FLAG, Pita^ΔCP1+2^-FLAG, and Pita^ΔCP1^-FLAG were expressed in transgenic flies at similar levels (Fig. 2c). The transgenes were crossed into the *pita*^*02132*^/*pita*^*k05606*^ background [24]. We confirmed the previously obtained result [26] that *pita*^*02132*^/*pita*^*k05606*^ heterozygotes do not express a detectable amount of the endogenous *pita* mRNA (Additional file 1). Unexpectedly, *Ubi-Pita*^*wt*^, *Ubi-Pita*^ΔCP1^, and *Ubi-Pita*^ΔCP1+2^ all complemented the *pita* mutations, which suggested that the CP190-interacting domains are not critical for the in vivo functions of the Pita protein.

To test the role played by the CP190-interacting domain in Pita in the recruitment of Pita and CP190 to chromatin, we compared the binding of CP190 and Pita to chromatin in *Ubi-Pita*^*wt*^ and *Ubi-Pita*^ΔCP1+2^ embryos. To identify the chromatin binding sites of CP190 and Pita-FLAG in embryos, we performed chromatin immunoprecipitation (ChIP) experiments, followed by sequencing (ChIP-seq) using Illumina’s massive parallel sequencing technology.

**Fig. 3:**
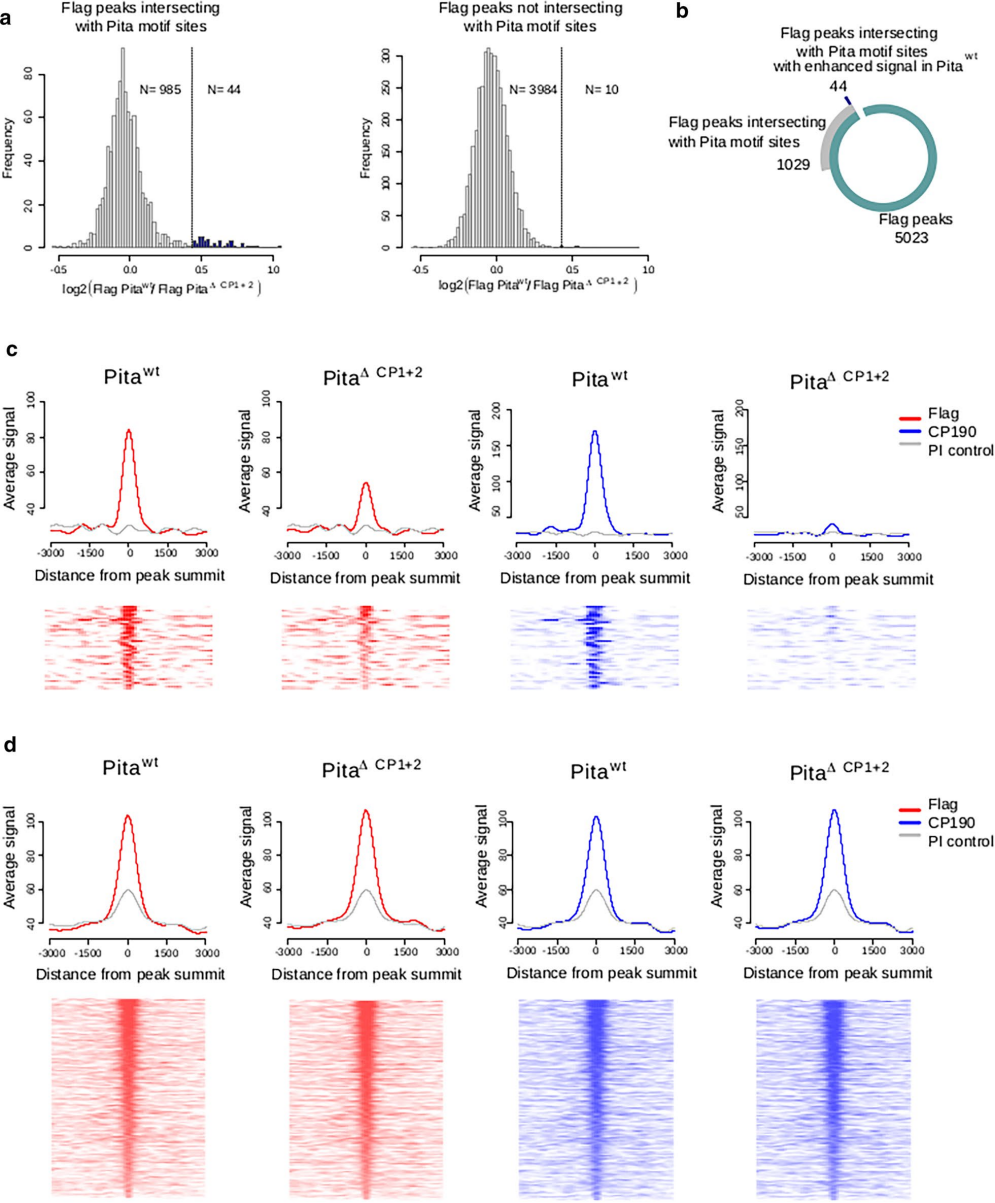
FLAG and CP190 ChIP-seq signal analysis for different sets of FLAG peaks. **a** The distribution of log fold changes between FLAG signals in Pita^*wt*^ and Pita^ΔCP1+2^ lines among the FLAG peaks that intersect (on the left) and do not intersect (on the right) with previously defined Pita motif sites [17] (see “Materials and methods”). Outliers of the distributions are colored in blue. Outlier peaks from the peak set that intersects with Pita motif sites (*N* = 44) were further analyzed as an independent peak set. **b** The numbers of peaks in the investigated peak sets. **c** Average signal (RPKM) (on the top) and signal heatmaps (on the bottom) for FLAG and CP190 signals among the FLAG peaks that intersect with Pita motif sites and demonstrate enhanced FLAG signal in Pita^wt^ (*N* = 44) (Group 1). On the heatmaps, the peaks are ranked according to the average FLAG signal in Pita^*wt*^ and Pita^ΔCP1+2^ lines. **d** Average signal (RPKM) (on the top) and signal heatmaps (on the bottom) for FLAG and CP190 signal among the FLAG peaks that intersect with Pita motif sites without enhanced FLAGlag signal in Pita^*wt*^ (*N* = 985) (Group 2). On the heatmaps, the peaks are ranked according to the average FLAG signal in Pita^*wt*^ and Pita^ΔCP1+2^ lines

To investigate changes in the chromatin binding of CP190 and Pita in the *Pita*^ΔCP1+2^ mutant, ChIP-seq signal values were estimated in the set of FLAG peaks reproduced in Pita^*wt*^ and Pita^ΔCP1+2^ embryos. We found 5023 such FLAG peaks (Fig. 3b). Then, we defined 1029 peaks that overlapped with the Pita motif site obtained from previously published data [17]. From among these 1029 peaks, we selected 44 peaks that demonstrated an enhanced signal in Pita^*wt*^ embryos compared with Pita^ΔCP1+2^ embryos (Fig. 3a). Among the 3994 FLAG peaks that did not intersect with Pita motif sites, we found only 10 peaks with enhanced signals in Pita^*wt*^ compared with Pita^ΔCP1+2^. As a result, the Pita^ΔCP1+2^ binding efficiency was only significantly reduced in a minor proportion of the binding sites. Thus, CP190 binding is not essential for Pita binding to most chromatin sites.

All Pita peaks were divided into three groups. In group 1, we included Pita motif site peaks with at least a twofold decrease in the average signal for Pita^ΔCP1+2^ embryos compared with that in Pita^*wt*^ embryos (Fig. 3c). Group 2 consisted of peaks with Pita motif sites in which no significant changes in the FLAG signals were observed when comparing the results of Pita^*wt*^ and Pita^ΔCP1+2^ embryos (Fig. 3d). All FLAG peaks that did not intersect with a Pita motif were included in Group 3 (Additional file 2A).

**Fig. 4:**
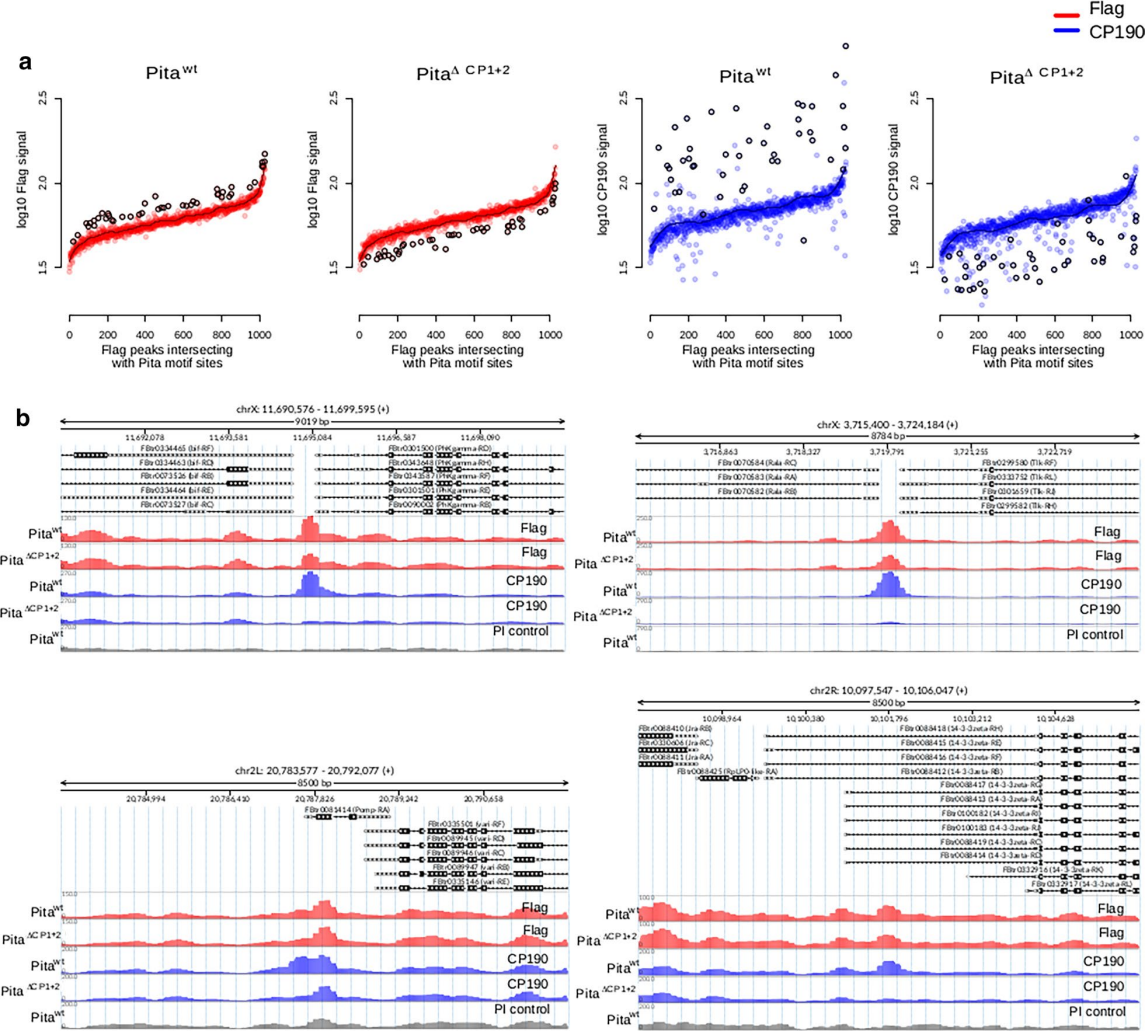
Flag and CP190 ChIP-seq signal depletion in the Pita^ΔCP1+2^ line. **a** Log_10_ of the average FLAG and CP190 signal (RPKM) in FLAG peaks that intersect with Pita motif sites (*N* = 1029), ranked according to the average FLAG signal in Pita^*wt*^ and Pita^ΔCP1+2^ lines. Peaks with enhanced FLAG signals in Pita^*wt*^ are marked with black circles (*N* = 44). The black line shows the average curve shape obtained in Pita^*wt*^ lines for FLAG and CP190 signals. **b** Examples of CP190 signal depletion in the Pita^ΔCP1+2^ line among FLAG peaks with and without FLAG signal depletion in the Pita^ΔCP1+2^ line (RPKM)

Then we compared the CP190 signal in these three groups of peaks. CP190 binding falls extremely low among the sites in Group 1 (Fig. 3c), whereas no visible changes were observed for the sites from Groups 2 (Fig. 3d) and 3 (Additional file 2A). The analysis of individual FLAG-binding sites showed that in Group 1 (Fig. 4a), in parallel with the twofold decrease in FLAG binding in Pita^ΔCP1+2^ compared with Pita^*wt*^, a significant decrease in CP190 binding occurred (Fig. 4b, top). At the same time, in Groups 2 (Fig. 4a) and 3 (Additional file 2B), on the background of stable FLAG binding, the partial weakening of CP190 binding was observed at several sites (Fig. 4b, bottom), although most sites demonstrated the maintenance of stable CP190 binding. We studied the co-localization of Pita binding sites with sites of other proteins (Additional file 3) that interact with CP190 [17, 25, 44–47]. About 40% of Pita + CP190 sites are also co-localized with sites for either of Su(Hw), Ibf1, Ibf2, Insv, ZIPIC, and dCTCF. Since many of DNA-binding proteins that interacted with CP190 have not yet been identified, we can predict the existence of additional DNA-binding proteins located close to the Pita binding sites, which are capable of attracting the CP190 protein through a similar mechanism, masking the effects of mutant Pita ^ΔCP1+2^.

### CP190-interacting domains in Pita are critical for the formation of the interband region in larvae polytene chromosome

Pita binding sites are typically located in the promoter regions and interbands of *Drosophila* polytene chromosomes [17, 25]. Recent studies showed that the interbands of polytene chromosomes typically correspond to the promoter regions of broadly expressed housekeeping genes and display an “open” chromatin conformation [48, 49]. Interbands have been reported to be preferentially associated with the CP190 and Chromator (Chrom/Chriz) proteins [46, 50, 51].

Because the linker region (94–285 aa) of Pita recruits CP190, we explored whether the linker region was sufficient for the organization of open chromatin. To address this question, we used a previously established model system based on *Drosophila* polytene chromosomes [52]. In this model, 14 GAL4 binding sites were inserted into the silent region 10A1-2. The *pita* gene region encoding the linker (94–285 aa) was fused in-frame with the DNA-binding domain of the yeast protein GAL4 (GAL4DBD), under the control of the hsp70 promoter. The expression vector was inserted into the 51C region on the second chromosome, using the φC31-based integration system [43]. The 10A1-2 insertion was combined with the hsp70_Pita[94–295]GAL4DBD construct. To express the chimeric protein, flies were maintained at 29 °C from the embryonic to pupal stages, as described in [52].

**Fig. 5:**
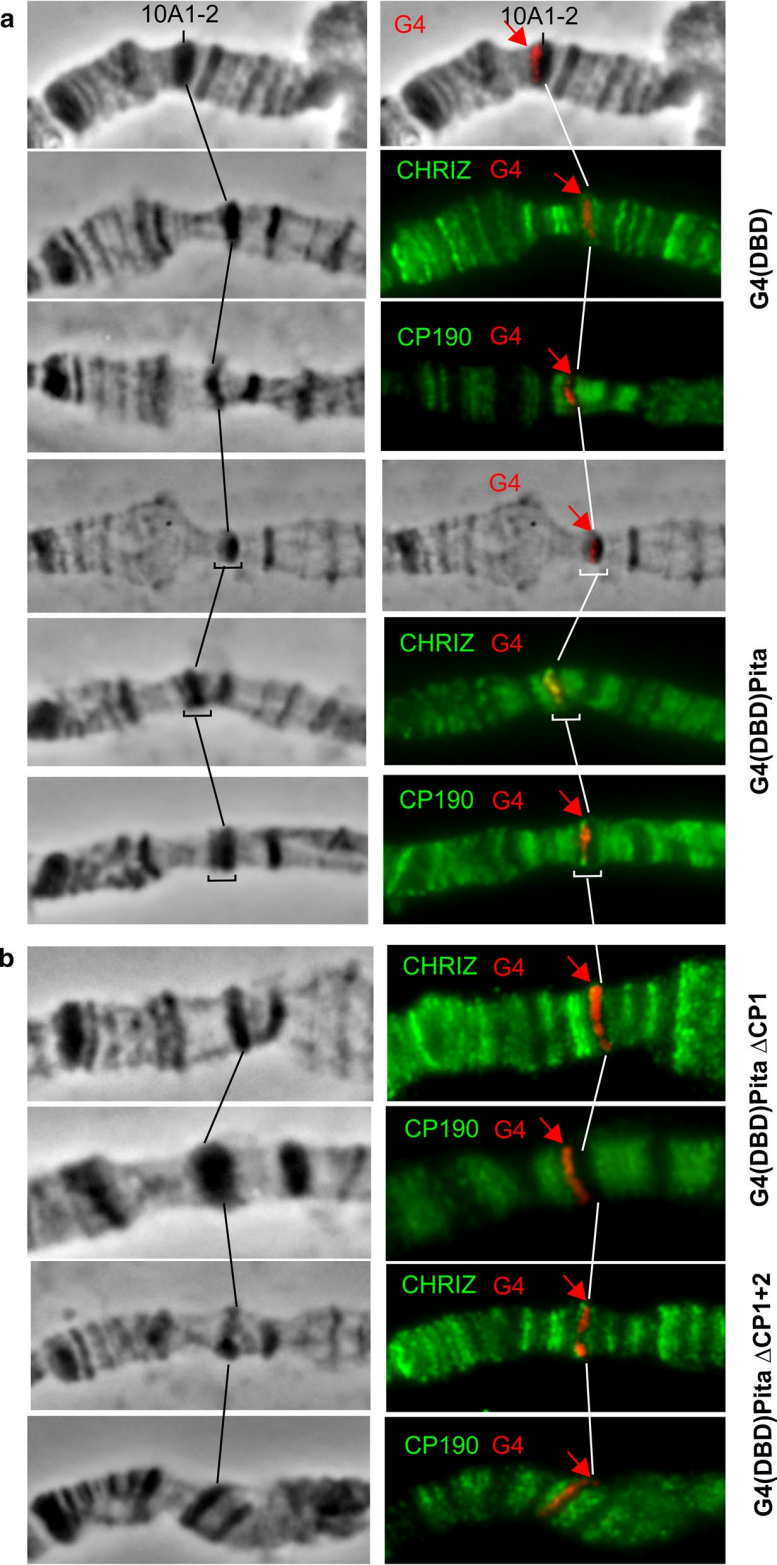
Testing the role played by the CP190-binding region of Pita to induce an “open” chromatin structure on a polytene chromosome model. The left panel demonstrates the polytene chromosomes in phase contrast. The right panel is an overlay of phase contrast and immunostaining with antibodies against to Gal4 (red), Chriz (green), and CP190 (green). **a** Targeting the 94–285 aa Pita region (Pita) fused with the GAL4 DNA-binding region (GAL4DBD) to the 16 GAL4 binding sites in the 10A1-2 disc. At the top, the recruitment of GAL4DBD did not induce the formation of the interband in the 10A1-2 band (negative control). At the bottom, the recruitment of the 94–285 aa Pita region fused with GAL4DBD (G4(DBD)Pita) resulted in interband formation inside the band, and Chriz and CP190 proteins are detected in the decompacted area (shown in brackets and arrows). **b** The recruitment of chimeric proteins featuring the deletion of CP1 (G4(DBD)Pita^ΔCP1^) or CP1 + CP2 (G4(DBD)Pita^ΔCP1+2^) regions to the 10A1-2 did not induce interband formation inside the disc. The absence of CP190 and Chriz protein recruitment was detected simultaneously with the presence of a signal for Gal4 at the compact disk structure (red arrow)

We used a previously described transgenic line [52], which expresses the GAL4 binding region under the control of the hsp70 promoter (G4(DBD)), as a negative control. In this line, the G4(DBD) is recruited to the 10A1-2 region, but does not change the polytene organization and fails to recruit CP190 (Fig. 5 a). The expression of Pita[94–295] (G4(DBD)Pita) gave rise to a prominently decondensed zone on the edge of 10A1-2 that split away from a distal part of the 10A1-2 band (Fig. 5 a). Thus, the recruitment of Pita[94–295] to the GAL4 sites was sufficient for interband formation. On polytene chromosomes, CP190 and Chriz co-localized with the decondensed region, suggesting that both proteins were recruited to the GAL4 sites by the Pita linker. As controls, we used the same model system to test Pita linkers featuring the deletion of either the 13 aa core (Pita[94–295]^ΔCP1^) or CP190-binding regions (Pita[94–295]^ΔCP1+2^) (Fig. 5b). For both deletions, we did not observe the formation of decondensed regions and or the recruitment of the CP190 and Chriz proteins. These results confirmed the role played by the 220–232 aa core region of Pita in the recruitment of CP190 and Chriz proteins and in chromatin opening.

### The deletion of the CP190-interacting domain in Pita affects the boundary functions of multimerized Pita sites in vivo

**Fig. 6:**
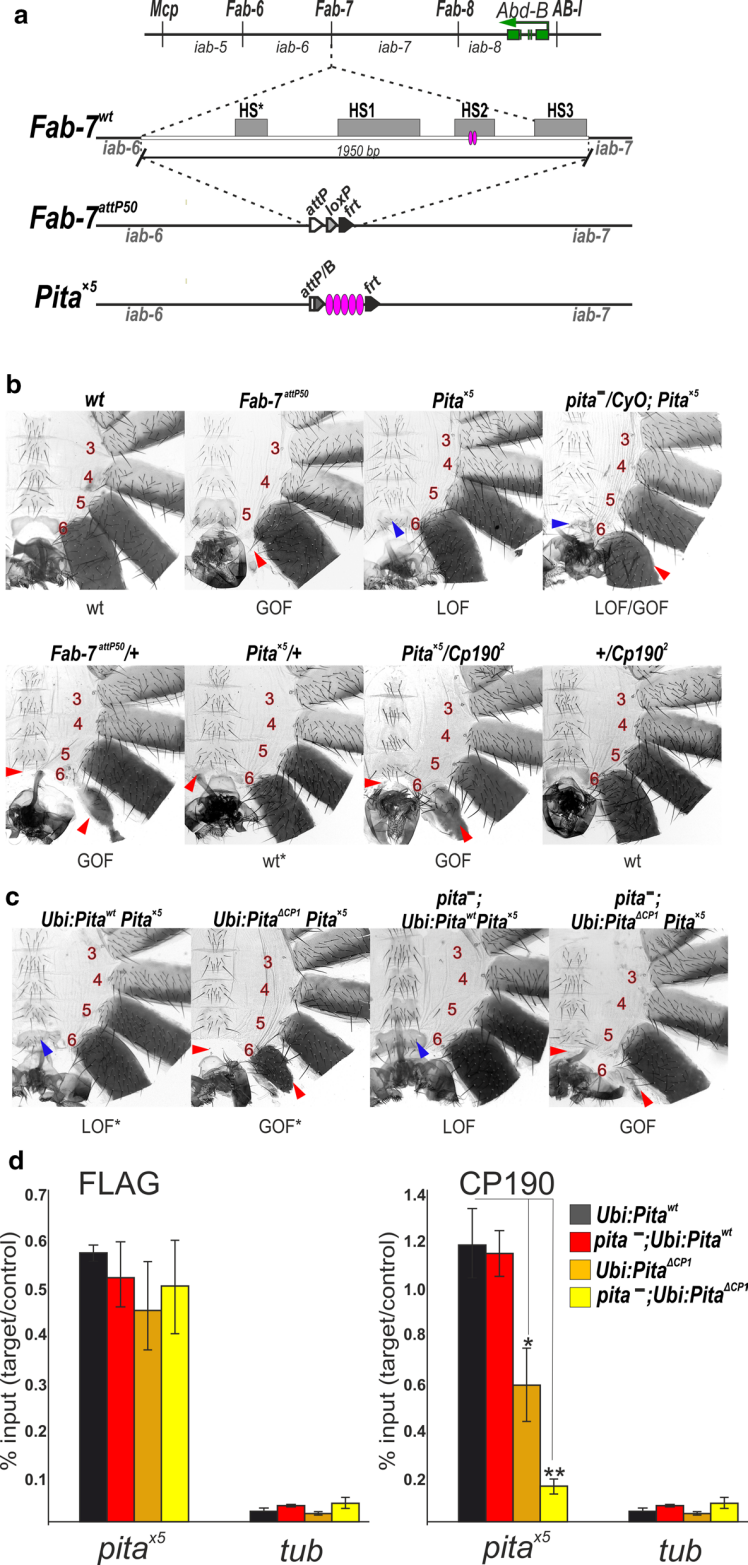
CP190 is required for Pita boundary activity. **a** A schematic showing the regulatory regions of the *Abd-B* gene. The green arrow indicates the *Abd-B* gene. The *iab*-domains (*iab-5–iab-8*) are separated by boundaries (*Mcp*, *Fab-6*, *Fab-7*, and *Fab-8*) that are shown by vertical black bars. Below, a schematic representation of the *Fab-7* boundary replacements at the *Fab-7*^*attP50*^ deletion. The HS*, HS1, HS2, and HS3 hypersensitive sites are indicated as grey boxes. The *Fab-7*^*attP50*^ deletion contains an *attP* site for transgene integration and *lox*- and *frt*-sites for the excision of the reporter genes and plasmid sequences. **b** The morphologies of abdominal segments (numbered) in males carrying different combinations of mutations. The red arrows show the signs of a gain-of-function (GOF) phenotype (transformation of the A6 segment into a copy of A7). The blue arrows show the signs of a loss-of-function (LOF) transformation (transformation of the A6 segment into a copy of A5) that is directly correlated with the boundary functions of tested DNA fragments. In *Fab-7*^*attP50*^ males, A6 transforms into A7 (GOF), which leads to the absence of a corresponding segment. In *wt* males, the A5 sternite has a quadrangular shape and is covered with bristles, whereas the A6 sternite has a distinctly concave, elongated shape and lacks bristles. In *Pita*^×*5*^ males, the A6 segment is transformed into a copy of A5: both sternites have a quadrangular shape and are covered with bristles. *pita*^*−*^/*CyO* and *pita*^*−*^ indicate *pita*^*k05606*^/*CyO* and *pita*^*02132*^/*pita*^*k05606*^, respectively. **c** Morphologies of the abdominal segments (numbered) in *Pita*^×*5*^ males expressing *Ubi:Pita*^*wt*^ or *Ubi:Pita*^*ΔCP1*^ in the *wild-type* or *pita*^*−*^ (*pita*^*02132*^/*pita*^*k05606*^) background. **d** Compared with the binding of FLAG-Pita and CP190, the binding region in males expressing *Ubi:Pita*^*wt*^ or *Ubi:Pita*^*ΔCP1*^ were assessed in the wild-type or *pita*^*−*^ background. Histograms show ChIP enrichments at the Pita^×5^ region on chromatin isolated from males expressing different variants (*wt* and lacking the CP190-binding region) of Pita protein. The results are presented as a percentage of input genomic DNA, normalized to the corresponding positive autosomal genome region at the 100C cytological locus. Error bars show standard deviations of triplicate PCR measurements for two independent experiments. Asterisks indicate significance levels: *p < 0.05, **p < 0.01

To test the functional role of the Pita-CP190 interaction in insulation, we used a model system (Fig. 6a) based on a transgenic line in which the *Fab-7* boundary has been replaced with five Pita binding sites (Pita^×5^) [19, 53]. The *Fab-7* boundary blocks cross-talk between the *iab-6* and *iab-7* regulatory domains, which, respectively, stimulate lower levels of *Abd-B* transcription in PS11 and higher levels in PS12 [31]. In *wt* cells in the A6 (PS11) and A7 (PS12), the abdominal segments have different fates in adult males. The A6 cells form distinct cuticular structures (tergites and sternites) and the internal tissues of the abdominal segment, whereas the A7 cells are lost during metamorphosis (Fig. 6b). In the absence of a boundary between these two domains (*Fab-7*^*attP50*^ mutant males), *iab-7* is ectopically activated in all A6 (PS11) cells, and they assume an A7 (PS12) identity. These males lack both the A6 and A7 segments (Fig. 6b). The insertion of the Pita^×5^ sites blocks the cross-talk between the *iab-6* and *iab-7* domains but does not allow for communications between the *iab-6* enhancers and the *Abd-B* promoter. As a result, the *iab-5* enhancers stimulate the *Abd-B* transcription in A6, which results in the conversion of the A6 segment into one that resembles the A5 segment (Fig. 6b). Decreasing the protein level by half due to the introduction of the Pita mutation leads to the loss of the insulating function of the Pita^×5^ boundary in some cells, which is reflected by the reduction and deformation of the A6 sternite (Fig. 6b).

Heterozygous *Pita*^×*5*^/+ males display a very weak A6 → A5 transformation, suggesting that Pita^×5^ can block the cross-talk between the *iab-6* and *iab-7* regulatory domains (Fig. 6b). However, *Pita*^×*5*^/+ males that also carry heterozygous null mutations in the *Cp190* gene, *Cp190*^2^*/*+ *or Cp190*^3^/+ [54], display the partial transformation of A6 into a copy of A7 (Fig. 6b). The equally high sensitivity to mutations in the *Cp190* and *pita* genes suggests that CP190 acts as a key factor in the organization of the Pita-mediated boundary.

Next, we combined one copy of the *Ubi-Pita*^*wt*^ or *Ubi-Pita*^ΔCP1^ with *Pita*^×*5*^ (Fig. 6c). In contrast with Pita^*wt*^-FLAG, the overexpression of Pita^ΔCP1^-FLAG led to a partial transformation of A6 towards A7 (Fig. 6c). To test changes in the binding of Pita variants and CP190 with the *Pita*^×*5*^ region, we used the quantitative analysis of ChIP (ChIP-qPCR) performed in extracts obtained from adult 3-day-old males (Fig. 6d). Anti-FLAG antibodies were used to test the over-expressed Pita variants. The ChIP study showed that Pita^wt^-FLAG and Pita^ΔCP1^-FLAG bound with similar efficiency to the *Pita*^×*5*^ region. In contrast, the binding of CP190 to the *Pita*^×*5*^ region was reduced in a transgenic line expressing Pita^ΔCP1^. Thus, boundary activity mediated by Pita^×5^ was closely correlated with the efficiency of attracting CP190 to this region.

To directly demonstrate the role played by the CP190–Pita interaction during boundary activity, we constructed transgenic lines homozygous for Pita^×5^ and either the *Ubi-Pita*^*wt*^ or *Ubi-Pita*^ΔCP1^ transgenes in the *pita*^*02132*^/*pita*^*k05606*^ background. Pita^*wt*^ supported the boundary activity of the *Pita*^×*5*^ region (Fig. 6c). In contrast, the expression of Pita^ΔCP1^ led to an almost complete loss of boundary activity for the Pita^×5^ region (the absence of the A6 segment). In the ChIP analysis, Pita^wt^-FLAG and Pita^ΔCP1^-FLAG both bound to the *Pita*^×*5*^ region with similar efficiencies (Fig. 6d). CP190 was only observed at the Pita^×5^ sites in the transgenic line expressing Pita^*wt*^. These results confirmed that the 13 aa core is essential for the binding between CP190 and the Pita sites and that CP190 is essential for the boundary activity of Pita.

## Discussion

In this study, we mapped the regions of the Pita and CP190 proteins that are involved in their interaction. The interaction primarily occurs between the 13 aa core (CP1) of Pita and the BTB domain of CP190. The Pita 114–164 aa (CP2) region plays only an auxiliary role in the interaction, which might stabilize the CP190–Pita complex on chromatin. The knockdown of CP190 in *Drosophila* cell lines was previously found to affect Su(Hw) binding but not dCTCF binding [46]. Here, we demonstrated that the interaction with CP190 is required only for the binding of Pita to a small region of the chromatin site. We did not observe any differences in the binding of Pita^*wt*^ and Pita^ΔCP1^ to the Pita^×5^ sites. Moreover, the mutant protein can effectively compete with the wild-type analog to bind with the Pita^×5^ sites.

In polytene chromosomes, interbands appear as decondensed regions that coincide with the promoters of housekeeping genes and TAD boundaries [50, 52, 55–57]. The constant decondensation of interband regions is a consequence of nucleosome destabilization, the appearance of open chromatin sites, and the binding of transcription factors. Here, we demonstrated that the 13 aa region (CP1) of the Pita 94–295 aa linker is critical for the efficient recruitment of CP190 to the 14 GAL4 binding sites located in the condensed region of the 10A1-2 band. The recruitment of CP190 induces the decondensation of the region and the formation of the new interband. We found that CP190 can recruit the Chromator (Chrom/Chriz) protein, which is associated with all interband of polytene chromosomes [50, 51]. Currently, the role played by Chriz during chromatin organization is unknown; however, Chriz and CP190 may be involved in the recruitment of complexes participated in nucleosome remodeling and chromatin modifications. For example, experimental evidence has suggested that CP190 is involved in the recruitment of nucleosome remodeling factor (NURF), the Spt–Ada–Gcn5–acetyltransferase (SAGA) complex, the dimerization partner, RB-like, E2F, and multi-vulval class B (dREAM) complex, and the histone methyltransferase dMes4 [58–62]. Further study remains necessary to understand the role played by CP190 in the recruitment of different complexes involved in the organization of open transcriptionally active chromatin.

The architectural proteins Pita, Su(Hw), and dCTCF are involved in organization of boundaries/insulators in the BX-C [20]. When placed in the context of *Fab-7*, multimerized Pita-binding sites insulate the interaction between the active *iab-6* regulatory domain and the inactive *iab-7* regulatory domain, blocking thereby the premature activation of the *iab-7* domain in the A12 parasegment.

Our results showed that even the partial reduction of CP190 recruitment strongly affected the boundary activities of the Pita sites, suggesting a critical role played by CP190 in Pita-mediated insulation. The mechanism associated with CP190-dependent insulation remains unknown. CP190 might be involved in the formation of chromatin loops via interactions with Chriz [63]. Alternatively, CP190, Chriz, or other proteins recruited to the Pita sites may directly interfere with the ability of the initiators to interact functionally. Direct protein–protein interactions may be used to block the active signals from the *iab-6* to *iab-7* domain. Further research is needed to address this issue.

Although almost complete inactivation of Pita leads to lethality at the larval stage, the mutant Pita^ΔCP1^ and Pita^ΔCP1+2^ proteins, which failed to interact with CP190, had no discernable effects on fly viability. Thus, interactions with CP190 are not critical for the primary function of Pita during transcriptional regulation. The Pita mutants that lack the ability to recruit CP190 remained capable of binding DNA efficiently and support specific distance interactions through the ZAD domain, which is capable of homodimerization. Our recent model suggested that regulatory elements contain different combinations of binding sites for architectural proteins [21]. For example, Pita and dCTCF sites form the *Mcp* boundary between the *iab* domains that are involved in the regulation of the *abd-A* and *Abd-B* genes [19, 64]. The binding of dCTCF to *Mcp* is highly dependent on the presence of the Pita site, suggesting that Pita may function to assist the binding of other architectural proteins to regulatory elements. The inability of Pita to interact with CP190 is likely compensated by other architectural proteins that cooperate with Pita in the organization of the same regulatory regions. Indeed, we observed that CP190 still binds to most genomic sites associated with the Pita^ΔCP1+2^ protein in embryos. In many cases, these sites are associated with proteins that are known to be able to recruit CP190 [18, 25, 39, 40, 45, 65–67]. Such functional redundancy creates a stable and reliable architecture of regulatory elements, which is necessary for the correct regulation of genes during development.

## Materials and methods

### Pull-down assay

GST-pull-down was performed with immobilized glutathione Agarose (Pierce) in buffer C (20 mM Tris–HCl, pH 7.5; 150 mM NaCl, 10 mM MgCl_2_, 0.1 mM ZnCl_2_, 0.1% NP40, 10% (w/w) glycerol). BL21 cells co-transformed with plasmids expressing GST-fused derivatives of Pita and 6×His-thioredoxin-fused CP190[1–126] were grown in LB media to an A600 of 1.0 at 37 °C and then induced with 1 mM IPTG at 18 °C overnight. ZnCl_2_ was added to final concentration 100 μM before induction. Cells were disrupted by sonication in 1 mL of buffer C, after centrifugation lysate was applied to pre-equilibrated resin for 10 min at + 4 °C; after that, resin was washed four times with 1 mL of buffer C containing 500 mM NaCl, and bound proteins were eluted with 50 mM reduced glutathione, 100 mM Tris, pH 8.0, 100 mM NaCl for 15 min. 6×His-pulldown was performed similarly with Zn-IDA resin (Cube Biotech) in buffer A (30 mM HEPES–KOH pH 7.5, 400 mM NaCl, 5 mM β-mercaptoethanol, 5% glycerol, 0.1% NP40, 10 mM imidazole) containing 1 mM PMSF and Calbiochem Complete Protease Inhibitor Cocktail VII (5 μL/mL), washed with buffer A containing 30 mM imidazole, and proteins were eluted with buffer B containing 250 mM imidazole (20 min at + 4 °C).

### Plasmid construction

For in vitro experiments, protein fragments were either PCR-amplified using corresponding primers, or digested from Pita or CP190 cDNA and subcloned into pGEX-4T1 (GE Healthcare) or into a vector derived from pACYC and pET28a(+) (Novagen) bearing p15A replication origin, Kanamycin resistance gene, and pET28a(+) MCS.

To express 3×FLAG-tagged Pita and CP190 in the S2 cells, protein-coding sequences were subcloned into the pAc5.1 plasmid (Life Technologies). Different full-sized variants of Pita were fused with 3×FLAG and cloned into an expression vector. This vector contains *attB* site for φC31-mediated recombination, *Ubi-p63E* promoter with its 5′UTR, 3′UTR with SV40 polyadenylation signal, intron-less *yellow* gene as a reporter for detection of transformants. Details of the cloning procedures, primers, and plasmids used for plasmid construction are available upon request.

### Co-immunoprecipitation assay

*Drosophila* S2 cells were grown in SFX medium (HyClone) at 25 °C. S2 cells grown in SFX medium were co-transfected by 3×FLAG-Pita (wild-type and with deletion of CP190-interacting region) and CP190 plasmids with Cellfectin II (Life Technologies), as recommended by the manufacturer. Protein extraction and co-immunoprecipitation procedure were performed as described in [17]. Anti-CP190 antibodies and rat IgG were used for co-immunoprecipitations. The results were analyzed by Western blotting. Proteins were detected using the ECL Plus Western Blotting substrate (Pierce) with anti-FLAG and anti-CP190 antibodies.

### Fly crosses and transgenic lines

*Drosophila* strains were grown at 25 °C under standard culture conditions. The transgenic constructs were injected into preblastoderm embryos using the φC31-mediated site-specific integration system at locus 86Fb [43]. The emerging adults were crossed with the *y ac w*^*1118*^ flies, and the progeny carrying the transgene in the 86Fb region were identified by *y*^+^ pigmented cuticle. Details of the crosses and primers used for genetic analysis are available upon request.

### Fly extract preparation

20 adult flies were homogenized with a pestle in 200 μL of 1×PBS containing 1% β-mercaptoethanol, 10 mM PMSF, and 1:100 Calbiochem Complete Protease Inhibitor Cocktail VII. Suspension was sonicated 3 times for 5 s at 5 W. Then, 200 μL of 4×SDS-PAGE sample buffer was added and mixture was incubated for 10 min at 100 °C and centrifuged at 16,000×*g* for 10 min.

### RNA isolation and real-time PCR

Total RNA was isolated using the TRI reagent (Molecular Research Center, United States) according to the manufacturer’s instructions from larva, pupa and adult flies. RNA was treated with two units of Turbo DNase I (Ambion) for 30 min at 37 °C to eliminate genomic DNA. The synthesis of cDNA was performed using 2 µg of RNA, PrimeScript reverse transcriptase (Takara), and oligo(dT) as a primer. The amounts of specific cDNA fragments were quantified by real-time PCR. At least three independent measurements were made for each RNA sample. Relative levels of mRNA expression were calculated in the linear amplification range by calibration to a standard genomic DNA curve to account for differences in primer efficiencies. Individual expression values were normalized with reference to RpL32 mRNA.

The sequences of primers used in this work:

- 1 (pita ORF)—5′-gccacattgccactatca-3′ and 5′-ctgaacaagtcctcgattagg-3′;
- 2 (pita 3′UTR)—5′-aaaggccttcggttaaagg-3′ and 5′-agtgcatccgtgcttatg-3′;
- RpL32—5′-gttcgatccgtaaccgatgt-3′ and 5′-ccagtcggatcgatatgctaa-3’.

### Immunostaining of polytene chromosomes

Salivary glands were dissected from third-instar larvae reared at 29 °C. Polytene chromosome staining was performed as described [52]. The following primary antibodies were used: rabbit anti-CP190 (1:150), rabbit anti-Chriz (1:600). 3–4 independent staining, and 4–5 samples of polytene chromosomes were performed with each Pita-expressing transgenic line.

### ChIP-qPCR analysis

Chromatin for subsequent immunoprecipitations was prepared from adult flies as described in [25] with some modifications. Aliquots of chromatin were incubated with mouse anti-FLAG (1:200), rat anti-CP190 (1:500) antibodies or with nonspecific IgG purified from mouse and rat (control). At least two independent biological replicas were made for each chromatin sample. The enrichment of specific DNA fragments was analyzed by real-time PCR using a QuantStudio 3 Cycler (Applied Biosystems). The results of chromatin immunoprecipitation are presented as a percentage of input genomic DNA after triplicate PCR measurements. The *tub* coding region (devoid of binding sites for the test proteins) was used as a negative control; *100C* region was used as positive control. The sequences of used primers are available on request.

### ChIP-Seq analysis

Embryo collection and ChIP were performed as previously described [68]. Briefly, embryos were collected at 8–16 h and fixed with formaldehyde. Chromatin was precipitated with mouse anti-FLAG (1:100), anti-CP190 (1:200) antibodies, or with nonspecific mouse IgG. The ChIP-seq libraries were prepared with NEBNext® Ultra™ II DNA Library Prep kit, as described in the manufacturer’s instructions. Amplified libraries were quantified using fluorometry with DS-11 (DeNovix, United States) and Bioanalyzer 2100 (Agilent, United States). Diluted libraries were clustered on a pair-read flowcell and sequenced using a NovaSeq 6000 system (Illumina, United States). Raw and processed data were deposited in the NCBI Gene Expression Omnibus (GEO) under accession number GSE160007 (https://www.ncbi.nlm.nih.gov/geo/query/acc.cgi?acc=GSE160007).

ChIP-seq analysis was performed for 4 samples (FLAG and CP190 in Pita^*wt*^ and Pita^ΔCP1+2^ lines); two biological replicates were obtained for each sample. Paired-end sequencing technology was applied, with an average read length of 101. Adapters, poly-N, and poly-A read ends were removed using cutadapt software [69]. Cutadapt was also used to trim low-quality ends (quality threshold was set to 20 and reads with lengths less than 20 bp after trimming were discarded). The remaining reads were aligned against version dm6 of the *Drosophila melanogaster* genome using Bowtie version 2 [70]. Only reads that aligned concordantly exactly one time were passed for further analysis. The average insert size between mates was 156 bps. After alignment, read duplicates were removed using the Picard MarkDuplicates function (http://broadinstitute.github.io/picard/). Peaks that overlapped with blacklist regions were discarded (blacklist regions were previously converted from the dm3 to the constructed dm6 genome (https://sites.google.com/site/anshulkundaje/projects/blacklists). Peak calling was performed using MACS version 2 against a preimmune control [71], in paired-end mode (option format = BAMPE). Peaks with p-values less than 1 × 10^–2^ were passed to the irreproducible (IDR) pipeline to assess the reproducibility of ChIP-seq replicates (https://sites.google.com/site/anshulkundaje/projects/idr). All samples showed ideal or acceptable reproducibility status with a 0.05 IDR, *p*-value threshold [both the Rescue Ratio (RR) and the Self-consistency Ratio (SR) was less than 2, see Additional file 4)] (https://www.encodeproject.org/data-standards/terms/#concordance). An optimal set of reproduced peaks was chosen for each sample for further analysis. To ensure the comparability of signals in defined peaks comparable, the peak boundaries were defined as ± 250 bp from the peak summit for all further analyses. ChIP-seq coverage tracks (BedGraph) were obtained using deepTools [72], bamCoverage function with bin-width 100 bp, and the normalization of and reads per kilobase of transcript, per million mapped reads (RPKM).

To investigate the changes in CP190 and FLAG binding activity after Pita modifications, their ChIP-seq signal values were estimated in the set of FLAG peaks reproduced in Pita^*wt*^ and Pita^ΔCP1+2^ lines. To address the non-specificity of FLAG binding, this peak set was additionally divided according to the Pita motif appearance in the region ± 250 bp from the peak boundaries. The peaks intersecting with the Pita motif site were defined using SPRy-SARUS software (https://github.com/autosome-ru/sarus), with 10^–4^ *p*-value threshold. PWM (Additional file 5) was obtained by re-analysis of previously published data [17]: peak calling was performed as described above and then PWM was identified with ChIPMunk [73]. Additionally, from the peak set that intersects with Pita motif sites, we selected a number of peaks for which we observed enhanced signals in Pita^*wt*^ lines compared to Pita^ΔCP1+2^ lines. The peaks containing enhanced signals were identified by applying the Grubbs outlier detection method to the distribution of log fold change values between the FLAG signals in Pita^*wt*^ and Pita^ΔCP1+2^ lines: log_2_(Flag Pita^wt^/Flag Pita^ΔCP1+2^). The Grubbs method for one outlier was iteratively applied, while the *p*-value for the detected upper outlier was less than 0.05 (http://ftp.uni-bayreuth.de/math/statlib/R/CRAN/doc/packages/outliers.pdf).

Further analysis was performed in R version 3.6.3 [74]. Co-localization analysis was performed using ChIPpeakAnno package version 3.20.1 [75]. Average signal calculation and heatmaps were constructed with the use of ChIPseeker package version 1.22.1 [76]. Genomic tracks were visualized by applying svist4get software [77].

## Supplementary Information

**Additional file 1.** Characterization of the *pita* mutants. **A** Schematic representation of *pita* gene showing the localization of P-element insertions in the *pita*^*02132*^ and *pita*^*k05606*^ mutations. The positions of the nucleotide base pairs are given in the top of panel. The *pita* coding region is indicated by yellow boxes. The 5′ and 3′UTRs are shown with grey boxes. The introns are indicated by lines. The P{lacw}Dcp-1[k05606] and P{PZ}Dcp-1[02132] insertions are indicated by triangles. Schemes of inserted constructs are shown at the bottom of the panel. Red arrows with “1” and “2” labels show the positions of primers used for quantitative analysis. **B** Histogram shows the relative amount of *pita* mRNAs extracted from larva, pupa, adult in *y*^*1*^*w*^*1*^ and *pita*^*02132*^*/pitak*^*05606*^; *Ubi-Pita*^*wt*^ (pita-; Ubi-Pita^wt^) fly lines. “1” is a region from the ORF of *pita* mRNA that is present in the endogenous *pita* gene and *Ubi-Pita*^*wt*^ construct. “2” is a region from 3′UTR of *pita* mRNA that is present only in the endogenous *pita* gene. The real-time PCR shows that the endogenous *pita* gene is expressed in the *y*^*1*^*w*^*1*^ but not in *pita*^*02132*^*/pitak*^*05606*^; *Ubi-Pita*^*wt*^ (pita-; Ubi-Pita^wt^) line.

**Additional file 2.** FLAG and CP190 ChIP-seq signal analysis among the FLAG peaks that did not intersect with Pita motif sites. **A** Average signal (RPKM) (on the top) and signal heatmaps (on the bottom) for FLAG and CP190 signals among the FLAG peaks that do not intersect with Pita motif sites (*N* = 3994) (Group 3). Heatmaps show the peaks ranked according to the average FLAG signal in Pita^*wt*^ and Pita^ΔCP1+2^. **B** Log_10_ of the average FLAG and CP190 signal (RPKM) among FLAG peaks that do not intersect with Pita motif sites (*N* = 3994), ranked according to the average FLAG signal in Pita^*wt*^ and Pita^ΔCP1+2^ lines. The black lines show the average curve shape obtained in Pita^*wt*^ lines for the FLAG and CP190 signals.

**Additional file 3.** Co-localization of Pita with other DNA-binding proteins that interact with CP190. The table shows the total number of Pita + CP190 and Pita only peaks detected in the *Pita*^*wt*^ line. These two groups of peaks were tested for co-localization with the dCTCF, Su(Hw), ZIPIC, Ibf1, Ibf2, and Insv peaks obtained from [17, 44–47].

**Additional file 4.** Reproducibility of Chip-seq experiments according to IDR pipeline.

**Additional file 5.** PWM of Pita motif obtained using previously published data [17].

## Data Availability

All data generated or analyzed during this study are included in this published article and its Additional files. Raw and processed data of ChIP-seq analysis were deposited in the NCBI Gene Expression Omnibus (GEO) under accession number GSE160007.
