# Supplementary figures and images for: Mechanism and functional role of the interaction between CP190 and the architectural protein Pita in Drosophila melanogaster
Source: Epigenetics Chromatin. 2021 Mar 22;14:16. doi: 10.1186/s13072-021-00391-x (PMC7983404; doi:10.1186/s13072-021-00391-x)

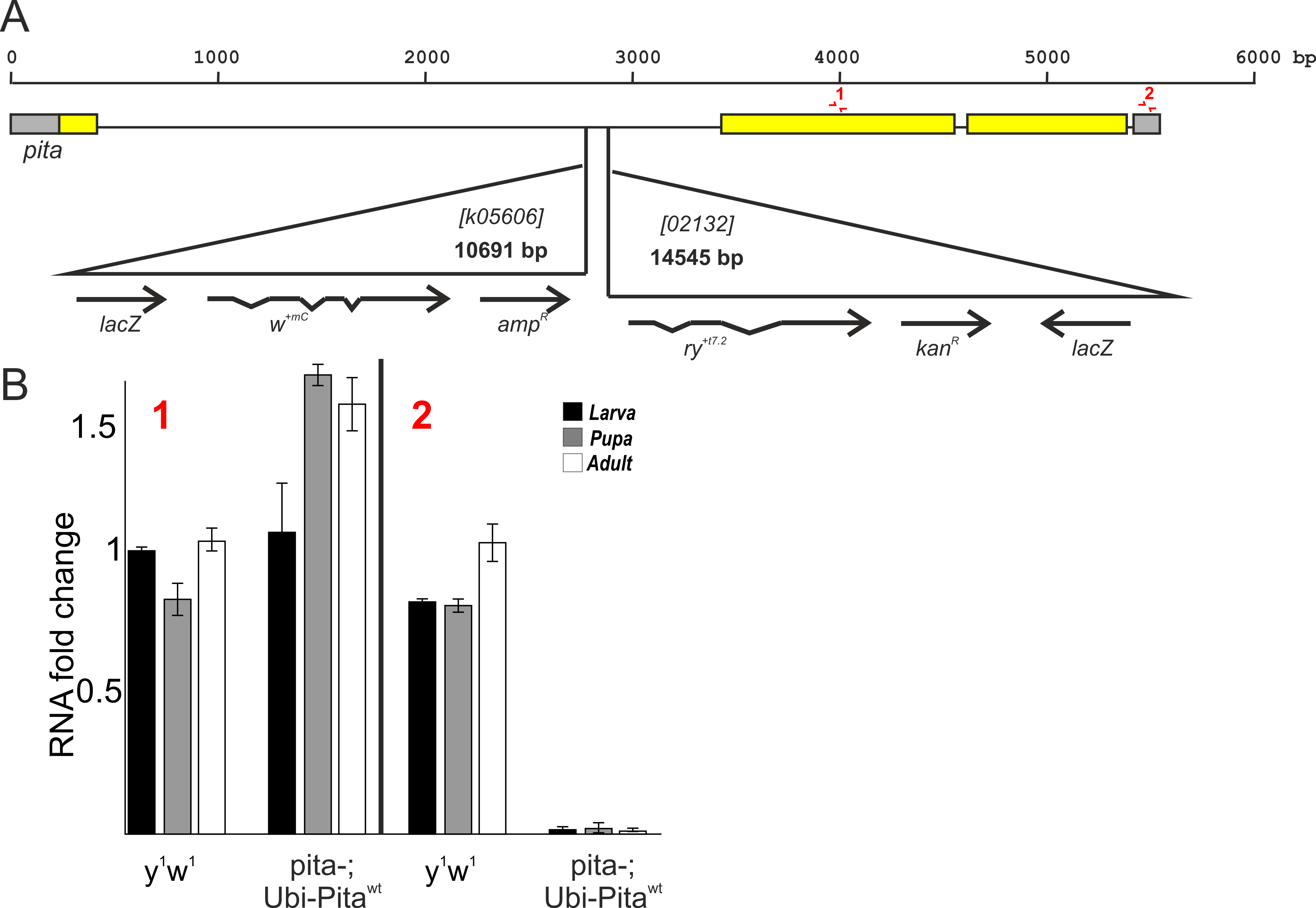

Supplement: Supplementary file 1 — Additional file 1. Characterization of the pita mutants. A Schematic representation of pita gene showing the localization of P-element insertions in the pita02132 and pitak05606 mutations. The positions of the nucleotide base pairs are given in the top of panel. The pita coding region is indicated by yellow boxes. The 5′ and 3′UTRs are shown with grey boxes. The introns are indicated by lines. The P{lacw}Dcp-1[k05606] and P{PZ}Dcp-1[02132] insertions are indicated by triangles. Schemes of inserted constructs are shown at the bottom of the panel. Red arrows with “1” and “2” labels show the positions of primers used for quantitative analysis. B Histogram shows the relative amount of pita mRNAs extracted from larva, pupa, adult in y1w1 and pita02132/pitak05606; Ubi-Pitawt (pita-; Ubi-Pitawt) fly lines. “1” is a region from the ORF of pita mRNA that is present in the endogenous pita gene and Ubi-Pitawt construct. “2” is a region from 3′UTR of pita mRNA that is present only in the endogenous pita gene. The real-time PCR shows that the endogenous pita gene is expressed in the y1w1 but not in pita02132/pitak05606; Ubi-Pitawt (pita-; Ubi-Pitawt) line. [file 13072_2021_391_MOESM1_ESM.tif]

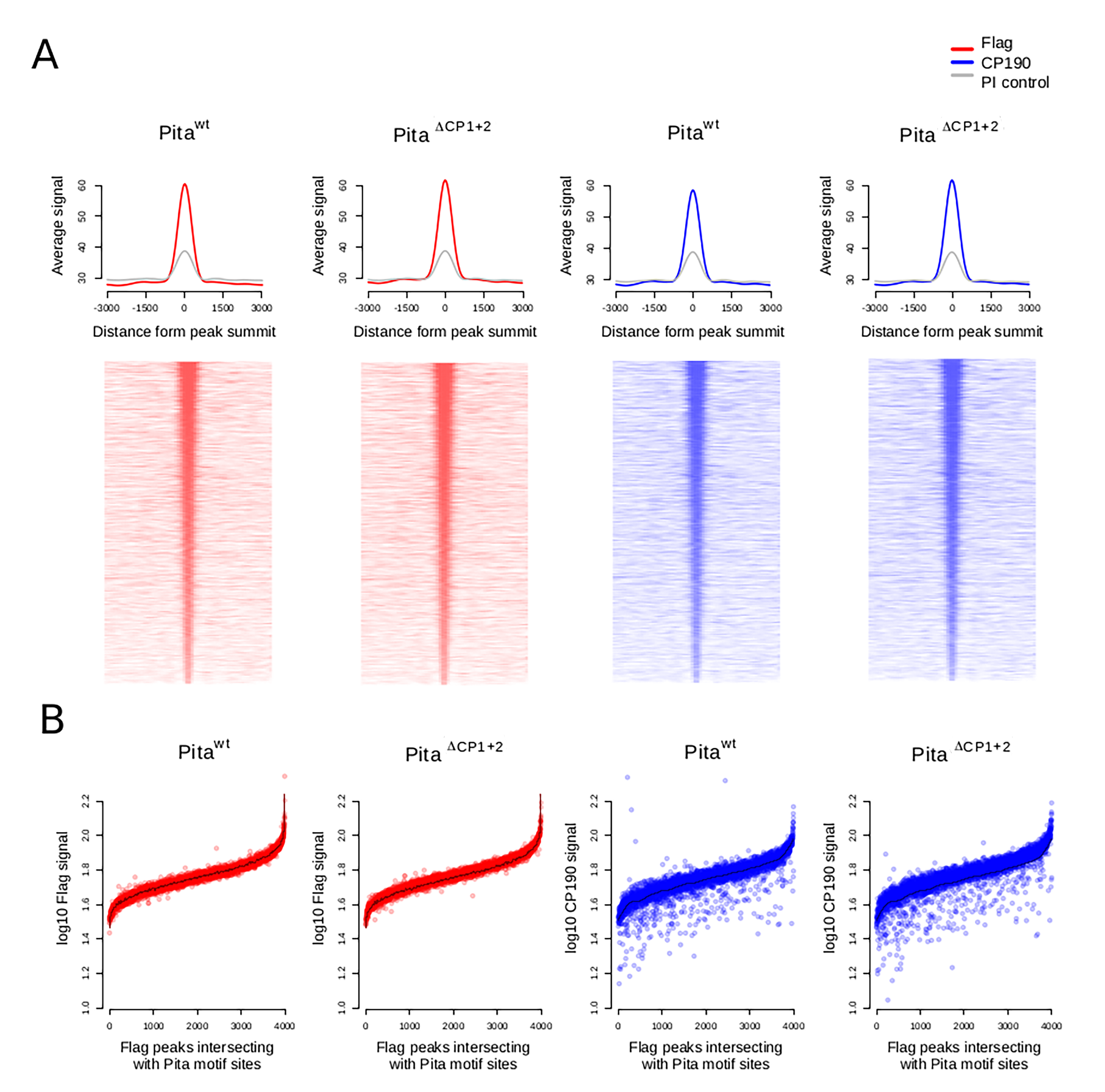

Supplement: Supplementary file 2 — Additional file 2. FLAG and CP190 ChIP-seq signal analysis among the FLAG peaks that did not intersect with Pita motif sites. A Average signal (RPKM) (on the top) and signal heatmaps (on the bottom) for FLAG and CP190 signals among the FLAG peaks that do not intersect with Pita motif sites (N = 3994) (Group 3). Heatmaps show the peaks ranked according to the average FLAG signal in Pitawt and PitaΔCP1+2. B Log10 of the average FLAG and CP190 signal (RPKM) among FLAG peaks that do not intersect with Pita motif sites (N = 3994), ranked according to the average FLAG signal in Pitawt and PitaΔCP1+2 lines. The black lines show the average curve shape obtained in Pitawt lines for the FLAG and CP190 signals. [file 13072_2021_391_MOESM2_ESM.tif]
