# Supplementary material for: Mechanism and functional role of the interaction between CP190 and the architectural protein Pita in Drosophila melanogaster
Source: Epigenetics Chromatin. 2021 Mar 22;14:16. doi: 10.1186/s13072-021-00391-x (PMC7983404; doi:10.1186/s13072-021-00391-x)
Supplement: Supplementary file 3 — Additional file 3. Co-localization of Pita with other DNA-binding proteins that interact with CP190. The table shows the total number of Pita + CP190 and Pita only peaks detected in the Pitawt line. These two groups of peaks were tested for co-localization with the dCTCF, Su(Hw), ZIPIC, Ibf1, Ibf2, and Insv peaks obtained from [17, 44–47]. [file 13072_2021_391_MOESM3_ESM.pdf]

Co-localization of Pita with other CP190-interacting proteins

	Pita	dCTCF	Su(Hw)	ZIPIC	Ibf1	Ibf2	Insv
Total	1029	587	2721	376	1686	1861	5476
Intersecting with Pita but not with CP190	160	1	12	1	4	4	15
Intersecting with Pita and CP190	869	15	80	14	95	97	169
