# Supplementary material for: Mechanism and functional role of the interaction between CP190 and the architectural protein Pita in Drosophila melanogaster
Source: Epigenetics Chromatin. 2021 Mar 22;14:16. doi: 10.1186/s13072-021-00391-x (PMC7983404; doi:10.1186/s13072-021-00391-x)
Supplement: Supplementary file 4 — Additional file 4. Reproducibility of Chip-seq experiments according to IDR pipeline. [file 13072_2021_391_MOESM4_ESM.pdf]

Reproducibility of Chip-seq experiments according to IDR pipeline

	RR	SCR	Number of peaks in optimal peak set
CP190 Pita$^{\Delta CP1+2}$	1.06	1.05	8429
CP190 Pita^{wt}	1.20	2.41	6586
Flag Pita$^{\Delta CP1+2}$	1.02	1.10	7365
Flag Pita^{wt}	1.18	2.66	6901
