# Supplementary material for: Mechanism and functional role of the interaction between CP190 and the architectural protein Pita in Drosophila melanogaster
Source: Epigenetics Chromatin. 2021 Mar 22;14:16. doi: 10.1186/s13072-021-00391-x (PMC7983404; doi:10.1186/s13072-021-00391-x)
Supplement: Supplementary file 5 — Additional file 5. PWM of Pita motif obtained using previously published data [17]. [file 13072_2021_391_MOESM5_ESM.pdf]

PWM of PITA motif obtained using previously published data [1]

A	C	G	T
0.02291816134	-0.04744336252	0.2684612076	-0.2115395592
-0.5293094702	0.4709414955	-0.3133820622	0.1807764722
-0.658301961	-0.3558135539	-0.4786430302	0.6509940678
-0.9298661471	-0.2996291108	-1.807197757	0.8356254389
1.136639017	-3.425853024	-3.425853024	-2.100138416
-2.972843551	-1.406117634	1.480535222	-1.352770603
-3.108286016	1.487420518	-4.40005804	-0.9298661471
-0.2949946296	0.4388283403	0.5615214231	-0.8662461235
0.3404021465	-0.7597424694	0.5672780668	-0.8064326419
0.602523982	-2.09051399	0.4646008353	-1.033595858
-3.108286016	-2.011812589	1.360796398	-0.4249335386
0.7100480396	-1.53839421	-0.4951143907	-0.3304296492
-2.15451591	1.334493822	-2.94188013	-0.3578550522
0.3898738906	-0.4307967895	-0.6767293315	0.06021400281
-1.015543032	1.256938341	-1.53839421	-0.6335771394
0.3170795947	0.32853355	0.32853355	-2.048565958
0.874208931	0.1709916456	-2.766385955	-2.482208618
1.019867774	-1.492330309	-2.09051399	-1.257220028
-3.264990487	1.607941206	-3.154869221	-3.976199186
-2.972843551	1.599798051	-3.798735508	-3.108286016
0.5174117349	-0.1715982172	-0.2996291108	-0.5293094702

1. Zolotarev N., Fedotova A., Kyrchanova O., Bonchuk A., Penin A.A., Lando A.S., Eliseeva I.A., Kulakovskiy I.V., Maksimenko O., Georgiev P. // Nucleic Acids Res. 2016. V. 44. № 15. P. 7228-7241.
